# Measuring robustness of brain networks in autism spectrum disorder with Ricci curvature

**DOI:** 10.1038/s41598-020-67474-9

**Published:** 2020-07-02

**Authors:** Anish K. Simhal, Kimberly L. H. Carpenter, Saad Nadeem, Joanne Kurtzberg, Allen Song, Allen Tannenbaum, Guillermo Sapiro, Geraldine Dawson

**Affiliations:** 1 Department of Electrical and Computer Engineering, Duke University, Durham, USA; 2 Department of Psychiatry and Behavioral Sciences, Duke Center for Autism and Brain Development, Duke University School of Medicine, Durham, USA; 3 Department of Medical Physics, Memorial Sloan Kettering Cancer Center, New York, USA; 4 Marcus Center for Cellular Cures, Duke University Medical Center, Durham, USA; 5 Brain Imaging and Analysis Center, Duke University, Durham, NC USA; 6 Department of Computer Science, Stony Brook University, Stony Brook, USA; 7 Department of Applied Mathematics and Statistics, Stony Brook University, Stony Brook, USA; 8 Department of Biomedical Engineering, Duke University, Durham, USA; 9 Department of Computer Sciences, Duke University, Durham, USA; 10 Department of Math, Duke University, Durham, USA; 11 Duke Institute for Brain Sciences, Duke University, Durham, USA

**Keywords:** Computational neuroscience, Autism spectrum disorders, Stem-cell research

## Abstract

Ollivier–Ricci curvature is a method for measuring the robustness of connections in a network. In this work, we use curvature to measure changes in robustness of brain networks in children with autism spectrum disorder (ASD). In an open label clinical trials, participants with ASD were administered a single infusion of autologous umbilical cord blood and, as part of their clinical outcome measures, were imaged with diffusion MRI before and after the infusion. By using Ricci curvature to measure changes in robustness, we quantified both local and global changes in the brain networks and their potential relationship with the infusion. Our results find changes in the curvature of the connections between regions associated with ASD that were not detected via traditional brain network analysis.

The prevalence of autism spectrum disorder (ASD) has been increasing over the past few decades. According to one recent study, almost 17% of children in the United States have been diagnosed with a neurodevelopmental disorder and approximately 2.5% are diagnosed with ASD^1^. ASD is clinically characterized by restricted interests and repetitive behaviors as well as social communication deficits^2^. Infants who later are diagnosed with ASD have atypical white matter developmental patterns compared to those typically developing infants and this difference is linked to the severity of ASD symptoms^3–5^. Previous research suggests that altered white matter development in ASD may result from neuroinflammation^6–11^. Autologous umbilical cord blood, a potential therapy, is theorized to reduce neuroinflammation^12,13^ and promote white matter development, thus triggering a reconfiguration of connectivity patterns in the brain^14^. In a previous paper from the same open-label clinical trial evaluating treatment with cord blood with young children with ASD, improvements in social functioning and communication abilities were described following treatment, which were correlated with an increase in connectivity of the white matter networks underlying the social and communicative functions^15,16^. These changes in both white matter volume and connectivity are usually measured via diffusion tensor imaging (DTI), a form of magnetic resonance imaging (MRI) which measures the diffusion of water molecules throughout the brain, a correlate for brain connectivity. While typical DTI analyses, such as those employing tractography measures, can provide usual information about white matter development in ASD, it does not account for all the connections between brain regions or examine the robustness of connections in the brain network. To take advantage of the network imaged by DTI, we need a measure that reflects a specific brain region’s relationship with every other region in the brain and that quantifies the robustness of such broad network connections. The aim of this work is to demonstrate the use of Ricci curvature to measure changes in robustness of white matter connectivity as imaged via DTI before and after a cord blood infusion.

Ricci curvature is a “measure by which a geometrical object deviates from being flat”^17^. Although there are multiple notions of graph curvature^18^, this work focuses on the Ricci curvature as formulated by Ollivier^19^, because of its positive correlation with the robustness of a network and because of its natural physical interpretation and computational efficiency. The link between curvature and robustness is as follows: curvature correlates positively with entropy; entropy correlates positively with robustness^17^. Robustness measures the extent to which a network can withstand perturbations. For brain networks, robustness measures the extent a region in a brain or a connection between two regions in the brain can be affected or withstand damage by a disease or a treatment.

The formal connection of curvature to robustness arises from several sources, including systems and control theory. Feedback tends to make a given system less sensitive, i.e., more robust, to parameter variations and external disturbances^20^. For a weighted graph derived from DTI, feedback is represented by the number of invariant triangles at a given node. Therefore, the greater the number of triangles^21^, the higher the curvature value. The curvature between two brain regions is computed by using a distance derived from the theory of optimal transport, and gives a novel measure of connectivity and feedback stability based on both local and global network geometry^19^. Thus, the curvature between two brain regions considers the strength of connection between those two brain regions in the context of the rest of the brain. This measurement takes into account the context of a brain region pair and serves as a useful lens through which to analyze the robustness of the brain.

To measure the safety and feasibility of cord blood infusions in children with ASD in an open-label clinical trial, nineteen participants were imaged via DTI and participated in a series of behavioral exams before and after the treatment. A full characterization of the sample is provided in^15^. The brain regions for each participant were delineated and defined as nodes of a network, while edges described structural connectivity between them. The DTI parcellation was done using the UNC Pediatric Brain Atlas. Ricci curvature was computed for the edges and a version of scalar curvature at the nodes by taking the weighted average of the Ricci curvature over all the neighboring edges. This is described in detail in the methods section. When analyzing the data, we looked at the change in behavioral scores and change in curvature. These changes were compared via Spearman correlation. The results presented are the changes in curvature between two nodes which correlate significantly ($\text{ p }<0.05$) with the change in behavior with two or more clinical tests. Potentially due to the relatively small data sample, none of the scalar (node) or edge (connection between ROIs) curvature correlations survived a false discovery rate (Benjamini–Hochberg correction^22^) with an alpha value of 0.05.

The results (Figure 1) highlight regions which have been previously indicated in ASD, but were not evident when constrained to the differences in white matter connectivity between pairs of individual brain regions^16^. In particular, using Ricci curvature, we see a relationship between clinical improvement and altered robustness in three white matter pathways that are implicated in the social and communication abilities that improved following treatment. The first novel connection for which we demonstrate a relationship between clinical improvement (as shown in Table 1) and increased curvature (robustness) is in a white matter pathway connecting the right dorsolateral prefrontal cortex (dlPFC) to the right insula. As shown in Table 2 and Figure 1, increased robustness within this pathway was correlated with improvements across all three clinical measures. Plots showing the correlation between the clinical scores and edge curvature are included in Supplemental Figure 1. Both of these regions have been implicated in autism^23,24^, with the insula in particular serving as a key structural and functional brain hub^25^. Resting state MRI (rsMRI) studies have suggested a role of the insula in one of the three canonical rsMRI networks, the salience network, which plays a critical role in detecting salient information from the sensory environment and engaging other functional networks, including the central-executive network of which the dlPFC is part^26^. The central-executive network is then responsible for integrating multiple cognitive processes, including working memory and attentional control, in the support of goal directed behaviors. Further, rsMRI studies of these networks have demonstrated that aberrant connectivity in these canonical networks correlate with social and communication abilities in children with ASD^27^. The two additional pathways for which we describe a novel relationship between clinical improvement and altered robustness were between the orbital frontal gyrus and the temporal cortex, as well as the rostral anterior cingulate and the hippocampus, both in the left hemisphere. These pathways both lie along major white matter tracts within the limbic system, namely the uncinate fasciculus and the cingulum, respectively^28^. Both of these pathways have demonstrated roles in social and communication abilities^29–31^ and have been previously implicated in autism^32–34^. Importantly and as previously mentioned, these three pathways did not show correlations with clinical improvement using canonical DTI analysis techniques as described in^16^, demonstrating the added value of using Ricci curvature.

In addition to identifying novel white-matter pathways showing a relationship between clinical improvement and alterations in robustness as measured via Ricci Curvature, we also show some concordance with findings from the more traditional DTI analyses. Specifically, we report a significant relationship between clinical improvement robustness between the right frontal pole and the inferior temporal gyrus, which lies along the uncinate fasciculus. The right uncinate fasciculus which is a major white matter pathway that has been implicated in emotion processing, memory, and language abilities^28,35,36^, has been shown to be related to decreased social and communicative abilities in individuals with autism^34, 37–40^, and was implicated in our previous study^16^. Additionally, we also find significant correlations between clinical improvement and altered metrics white matter connectivity in pathways involving the right basal ganglia using both classic DTI analyses and the novel Ricci Curvature described in this manuscript. The basal ganglia have been implicated in the pathophysiology of autism and may play a role in social motivation^41^. In both of the pathways within the uncinate fasciculus and the basal ganglia, the specific region pairs identified by Ricci curvature in the current study are not the same as those described using classic DTI tractography approaches as described in^16^. However, taken together these results support the right uncinate and the basal ganglia as a potentially important white matter pathway linked to improved social communication functioning in young children with autism.

Furthermore, the curvature analysis also identified regions for which the robustness of their connections with the rest of brain increased in relation to clinical outcomes, including the left fusiform gyrus, right pars orbitalis, left pericalcarine, and the left transverse temporal gyrus as shown in Table 3. Plots showing the correlation between the clinical scores and node curvature are included in Supplemental Figure 2. Both the pars orbitalis, which lies within the inferior frontal gyrus (IFG), and the fusiform gyrus are key components of the social brain network^42^. Previous research has linked the structure and function of both the fusiform gyrus and IFG to social cognition in autism^43,44^. The left pericalcarine and transverse temporal gyrus are components of the primary visual and auditory cortices, respectively. Both of these regions have been demonstrated to show anatomical differences that are associated with clinical functioning in autism^45–48^. Specifically, the left transverse temporal gyrus (aka Heschl’s gyrus) has been linked to language abilities in children with ASD^47^. Differences in the functional connectivity of the pericalcarine cortex with the frontal gyrus has been linked to higher levels of symptom impairment in both children and adolescents with autism^46^. Though preliminary due to the small sample size and fact that the trial was open-label, these results provide targets for investigation that will be explored in larger ongoing randomized, placebo-controlled trials.

**Table 1:** Changes in behavioral scores. Change in behavior is computed as the behavior measured at the end of the study minus the behavior measured at the beginning of the study. VABS-SS, Vineland Adaptive Behavior Scales-II Socialization Subscale; EOW, Expressive One-Word Picture Vocabulary Test; CGI-I, Clinical Global Impression Scale—Improvement.

	$\Delta$VABS-SS	$\Delta$EOW	$\Delta$CGI-I
Change in behavioral scores (mean ± SD)	3.37 ± 6.71	4.95 ± 6.34	2.79 ± 1.82

**Table 2:** Table of edge curvature results. Table showing the node pairs where the change in behavioral scores and change in curvature correlate with a $p<0.05$ for two or more behavioral exams. For each edge pair, the correlation between the change in curvature and change in behavioral score is listed, along with the associated p-value. The associated change in curvature is listed as mean ± standard deviation. Change in curvature is measured as ratio of the curvature of a node at the end of the study over the curvature of the node at the beginning of the study. VABS-SS: Vineland Adaptive Behavior Scales-II Socialization Subscale, EOW: Expressive One-Word Picture Vocabulary Test, CGI-I: Clinical Global Impression Scale - Improvement.

Edge pairs	VABS-SS	EOW	CGI-I	$\Delta$Curvature
R. Frontal Pole–R. Inferior Temporal	*r*: 0.575	*r*: 0.673	*r*: − 0.320	1.25 ± 1.49
	*p*: 9.96e−3	*p*: 1.60e−3	*p*: 1.82e−1	
R. Rostral Middle Frontal–R. Insula	*r*: 0.634	*r*: 0.656	*r*: − 0.583	1.14 ± 0.861
	*p*: 3.53e−3	*p*: 2.30e−3	*p*: 8.86e−3	
R. Bankssts–R. Accumbens Area	*r*: 0.638	*r*: 0.576	*r*: − 0.330	1.10 ± 0.458
	*p*: 3.30e−3	*p*: 9.85e−3	*p*: 1.68e−1	
R. Putamen–R. Pallidum	*r*: 0.590	*r*: 0.254	*r*: − 0.661	0.979 ± 0.106
	*p*: 7.79e−3	*p*: 2.93e−1	*p*: 2.08e−3	
L. Lateral Orbitofrontal Gyrus–L. Inferior Temporal Gyrus	*r*: 0.736	*r*: 0.367	*r*: − 0.646	1.83 ± 6.85
	*p*: 3.31e−4	*p*: 1.22e−1	*p*: 2.82e−3	
L. Rostral Anterior Cingulate–L. Hippocampus	*r*: 0.294	*r*: 0.623	*r*: − 0.607	0.901 ± 1.00
	*p*: 2.21e−1	*p*: 4.38e−3	*p*: 5.88e−3

**Table 3:** Table of scalar curvature results. Table showing the nodes where the change in behavioral scores and change in curvature correlate with a $p<0.05$ for two or more behavioral exams. For each node, the correlation between the change in curvature and change in behavioral score is listed, along with the associated p-value. The associated change in curvature is listed as mean ± standard deviation. Change in curvature is measured as ratio of the curvature of a node at the end of the study over the curvature of the node at the beginning of the study. VABS: Vineland Adaptive Behavior Scales-II Socialization Subscale, EOW: Expressive One-Word Picture Vocabulary Test, CGI: Clinical Global Impression Scale - Improvement.

Node	VABS-SS	EOW	CGI-I	$\Delta$Curvature
Right pars orbitalis	*r*: 0.651	*r*: 0.606	*r*: − 0.401	1.00 ± 0.060
	*p*: 2.53e−3	*p*: 5.94e−3	*p*: 8.85e−2	
Left pericalcarine	*r*: 0.581	*r*: 0.510	*r*: − 0.461	1.01 ± 0.083
	*p*: 9.14e−3	*p*: 2.55e−2	*p*: 4.70e−2	
Left fusiform	*r*: 0.577	*r*: 0.423	*r*: − 0.706	1.02 ± 0.121
	*p*: 9.68e−3	*p*: 7.14e−2	*p*: 7.27e−4	
Left transverse temporal gyrus	*r*: 0.498	*r*: 0.566	*r*: − 0.210	1.02 ± 0.072
	*p*: 3.01e−2	*p*: 1.15e−2	*p*: 3.88e−1	

These results must be considered in light of some limitations. Because this was an open label trial, it is not possible to determine whether the clinical and curvature changes were a result of normal trajectories of improvement and development or whether they were a consequence of the treatment itself. However, the current data provides targets for exploring brain-related changes in future randomized, placebo-controlled double-blind trials, which are currently taking place. Due to the number of DTI directions that were captured and the resolution of the data, cross hemisphere brain connections were removed. Future studies using higher-dimensional data are warranted.

Despite significant progress in understanding the underlying neurobiology of ASD, there are still few reliable and objective measures of change in social and communication function ASD and their relationship with underlying brain structures. We show that Ricci curvature identifies changes in robustness in brain regions that are correlated with improvements in social communication over time. Thus, this study lays the foundation for a new approach to assess both the robustness of a specific brain region and brain region pairs.

**Figure 1:**
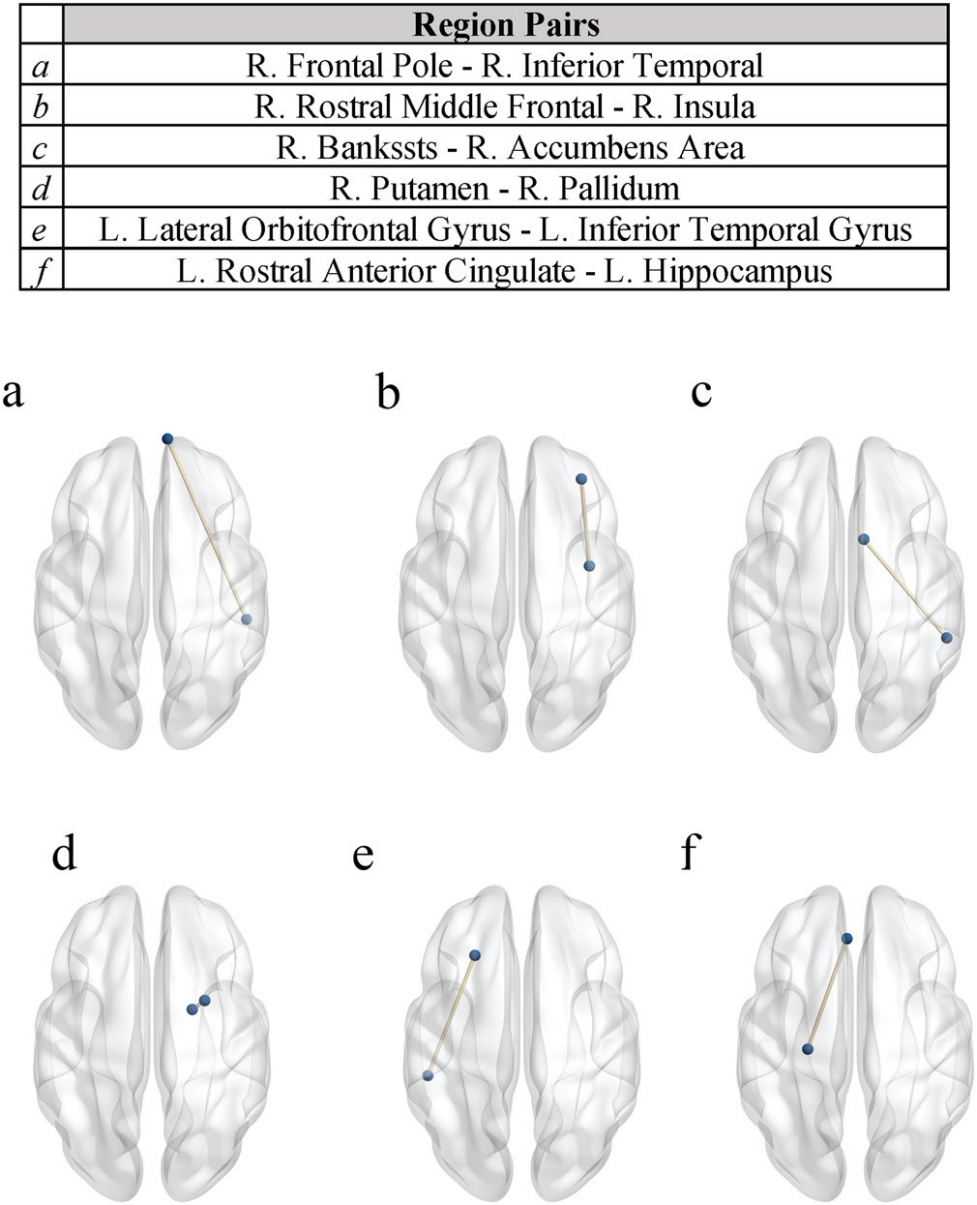
Overview of the results. Axial projection of pairs where the change in curvature correlated significantly with behavioral exams. Cross hemisphere brain connections were removed for this analysis. The brain graphics were visualized with the BrainNet Viewer (http://www.nitrc.org/projects/bnv/)^49^. L: left, R: right.

## Methods

### Study design and sample

The current study is a secondary data analysis of DTI data collected as part of a phase 1 open-label trial of a single intravenous infusion of autologous umbilical cord blood in 25 children with ASD who were between 24-72 months of age at baseline. The methods of this trial and the accompanying DTI analyses have been described in detail elsewhere^15,16,50,51^. Children with a confirmed diagnosis of ASD and a banked autologous umbilical cord blood unit of adequate size and quality participated in the trial. Nineteen participants provided high quality, artifact-free data for the DTI at both baseline and 6-month visits (17 males and 2 females). All caregivers/legal guardians of participants gave written, informed consent, and the study protocol was approved by the Duke University Health System Institutional Review Board. Methods were carried out in accordance with institutional, state, and federal guidelines and regulation. All methods and the trial were approved by the Duke Hospital Institutional Review Board and conducted under IND #15949. The ClinicalTrials.gov Identifier is NCT02176317.

### Clinical measures

The current study focuses on clinical measures for which clinical improvement was demonstrated in response to treatment^15^. Social abilities were measured with the Vineland Adaptive Behavior Scales-II Socialization Subscale (VABS-SS)^52^. The Vineland Adaptive Behavior Scale is a well-standardized parent report measure that yields an overall composite score of adaptive functioning, as well as subscale scores that include the socialization subscale. The VABS-SS was selected because of a priori hypotheses that treatment would impact social behavior in particular. Higher scores on the VABS-SS indicate better social functioning. The change in the VABS-SS (6 month-baseline) was used to measure change in social behavior. Expressive language was assessed with the Expressive One Word Picture Vocabulary Test 4 (EOW). The EOW is a clinician-administered assessment which measures an individual’s ability to match a spoken word with an image of an object, action, or concept^53^. Like the VABS-SS, higher scores on the EOW indicate better expressive language. The change in the raw score (6 month-baseline) was used to measure change in expressive language. Finally, clinical improvement was measured with the Clinical Global Impression Severity (CGI-S) and Improvement (CGI-I) scales^54^, which are commonly used rating scales that rate the children’s overall level of core ASD symptoms and related functioning and support requirements (CGI-S), as well as the amount of improvement or worsening of overall core ASD symptoms in addition to related functioning and need for supports from the time of the previous CGI-S rating (CGI-I). In the current study, the 6 month CGI-I rating was used to measure change in behavior between baseline and 6 month visits. Notably, lower scores on the CGI-I indicate more improvement.

### Magnetic resonance imaging acquisition and analysis

MRI scanning was conducted on a 3.0 T GE MR750 whole-body 60cm bore MRI scanner (GE Healthcare, Waukesha, WI). Participants were sedated to reduce motion artifacts in the MRI. Diffusion weighted images were acquired using a 25-direction gradient encoding scheme at $b = 1000\,\hbox {s/mm}^2$ with three non-diffusion-weighted images, an average (std) echo time (TE) of 85*m*s (2*ms*), and a repetition time (TR) of 12, 000*ms*. An isotropic resolution of $2\,\hbox {mm}^3$ was achieved using a $96 \times 96$ acquisition matrix in a field of view (FOV) of $192 \times 192\,\hbox {mm}^2$ at a 2 mm slice thickness. T1-weighted images were obtained with an inversion-prepared 3D fast spoiled-gradient-recalled (FSPGR) pulse sequence with a TE of 2.7*ms*, an inversion time (TI) of 450*ms*, a TR of 7.2*ms*, and a flip angle of $12^{\circ }$, at a $1\,\hbox {mm}^3$ isotropic resolution.

### Connectome analysis pipeline

The full connectome analysis pipeline is described in detail elsewhere^16^. Briefly, each participant’s T1 image and the first non-diffusion weighted image (b0) of the DTI acquisition were skull-stripped using the FSL brain extraction tool^55,56^. The T1 image was registered to the b0 image with an affine registration created using FSL FLIRT^57,58^. Region of interest (ROI) parcellation was performed by warping the dilated UNC Pediatric Brain atlas (available publicly at http://www.nitrc.org/projects/unc_brain_atlas/) into each participant’s T1 in diffusion image space via the Advanced Normalization Tools (ANTs) toolkit^59,60^. A total of 83 regions were defined for each participant, 41 gray matter regions in each hemisphere, and a single region encompassing the brainstem. FMRIB’s Automated Segmentation Tool (FAST) was used to calculate whole brain white matter volume for each participant at both baseline and 6 month visits^61^. Following this, a standardized pipeline for deterministic tractography based on the Connectome Mapper (CMP) was used to analyze participant data at both baseline and 6 month visits (http://www.cmtk.org)^14,62^. The parcellated gray matter ROIs included in this analysis are defined as nodes. Edges are defined as the volume of voxels containing valid streamlines that originate and terminate within a pair of nodes. For each participant, edge volumes were calculated and normalized by whole-brain white matter volume at both baseline and 6-month visits.

### Curvature analysis

In this section, we outline how we compute the Ricci curvature on discrete metric measure spaces including weighted graphs. The motivation of the Olliver-Ricci^19^ definition of curvature on a weighted graph is based on the following characterization of Ricci curvature from Riemannian geometry^63^. For *X* a Riemannian manifold, consider two very close points $x, y \in X$ and two corresponding small geodesic balls. Positive curvature is reflected in the fact that the distance between two balls is less than the distance between their centers. Similar considerations apply to negative and zero curvature. An increase in curvature corresponds to an increase in robustness which implies stronger pathways between nodes. When there is a strong correlation between improved behavioral scores and increased robustness, the implication could be that increased signal processing may be affecting behavioral outcomes.

For this work, the brain network is represented as an undirected and positively weighted graph, $G=(V, E)$, where *V* is the set of *n* vertices (nodes) in the network and *E* is the set of all edges (links) connecting them with weights $\{w\}$. Consider the graph metric $d:V\times V \rightarrow \mathbb {R}^+$ on the set of vertices *V* where *d*(*x*, *y*) is the number of edges in the shortest path connecting *x* and *y*. (*d* may be any intrinsic metric defined on *V*.) We let denote $w_{xy}>0$ denoting the weight of the edge between node *x* and *y*. (If there is no edge, then $w_{xy}=0.$) For any two distinct points $x, y \in V$, the Ollivier–Ricci (OR) curvature is defined as

1 $$\begin{aligned} k(x,y) := 1 - \frac{W_{1}(\mu _x,\mu _y)}{d(x,y)}, \end{aligned}$$

where $W_1$ denotes the Earth Mover’s Distance (Wasserstein 1-metric). We define the weighted degree at node *x*, $d_x$ as

2 $$\begin{aligned} d_x := \sum _z w_{xz}, \; \; \text{ the } \text{ sum } \text{ is } \text{ taken } \text{ over } \text{ all } \text{ nodes } z \text{ adjacent } \text{ to } x, \end{aligned}$$

and we define the probability measure at *x*, $\mu _x$ as

3 $$\begin{aligned} \mu _x(y) := \frac{w_{xy}}{d_x}, \; \; y \in V. \end{aligned}$$

The *scalar curvature* at a given node *x* (the contraction of Ricci curvature) is defined as

4 $$\begin{aligned} \hat{S}_{OR} (x) := \sum _{y} \mu _x(y) k(x,y), \end{aligned}$$

where the sum is taken over all neighbors of *x*. Given the discussed, positive correlation of robustness and curvature, in our work, we propose to use curvature as a proxy for robustness. Various advantages of using Ricci curvature in this framework are described in more detail in^17^. The code to compute curvature and to perform this analysis is shared at https://github.com/aksimhal/Curvature-ASD-Analysis.

### Statistical analysis

Correlations between changes in curvature and changes in behavioral scores were determined via Spearman correlation. Change in behavior is computed as the difference between the scores at the end of the study and the scores at the beginning of the study. Change in curvature is measured as ratio of the curvature of a node at the end of the study over the curvature of the node at the beginning of the study. The ratio of curvature is used instead of the difference because the curvature value has no inherent units associated with it to provide context about its meaning. For the results to be reported, the change in behavioral scores and change in curvature correlate with a $p < 0.05$ for two or more behavioral exams. Individual correlations between behavioral scores and change in curvature were examined using a false discovery rate (Benjamini–Hochberg) correction^22^ with an alpha value of 0.05.

## Electronic supplementary material

Supplementary material 1
