# Supplementary material for: Measuring robustness of brain networks in autism spectrum disorder with Ricci curvature
Source: Sci Rep. 2020 Jul 2;10:10819. doi: 10.1038/s41598-020-67474-9 (PMC7331646; doi:10.1038/s41598-020-67474-9)
Supplement: Supplementary file 1 — Supplementary material 1 [file 41598_2020_67474_MOESM1_ESM.pdf]

Measuring Robustness of Brain Networks in Autism Spectrum Disorder with Ricci Curvature

Anish K. Simhal[1, *, +], Kimberly L.H. Carpenter[2, +], Saad Nadeem[3, +], Joanne Kurtzberg[4], Allen Song[5], Allen Tannenbaum[6], Guillermo Sapiro[1, 7], and Geraldine Dawson[2, 4, 8]

[1]Department of Electrical and Computer Engineering, Duke University
[2]Duke Center for Autism and Brain Development, Department of Psychiatry and Behavioral Sciences, Duke University School of Medicine
[3]Department of Medical Physics, Memorial Sloan Kettering Cancer Center
[4]Marcus Center for Cellular Cures, Duke University Medical Center
[5]Brain Imaging and Analysis Center, Duke University, Durham, North Carolina
[6]Departments of Computer Science, Applied Mathematics and Statistics, Stony Brook University
[7]Departments of Biomedical Engineering, Computer Sciences, and Math, Duke University
[8]Duke Institute for Brain Sciences, Duke University
[*]aksimhal@gmail.com, corresponding author
[+]These authors contributed equally to this work

ABSTRACT

Ollivier-Ricci curvature is a method for measuring the robustness of connections in a network. In this work, we use curvature to measure changes in robustness of brain networks in children with autism spectrum disorder (ASD). In an open label clinical trials, participants with ASD were administered a single infusion of autologous umbilical cord blood and, as part of their clinical outcome measures, were imaged with diffusion MRI before and after the infusion. By using Ricci curvature to measure changes in robustness, we quantified both local and global changes in the brain networks and their potential relationship with the infusion. Our results find changes in the curvature of the connections between regions associated with ASD that were not detected via traditional brain network analysis.

Supplemental Curvature Information

Introduction

Geometric techniques can be used to study the properties of complex networks, including as a method to measure a network's robustness to certain perturbations [1, 2, 3, 4, 5]. Curvature, for this task, is based on the theory of optimal mass transport [6, 7]. First, note that the space of probability densities on a given Riemannian manifold also inherits a natural Riemannian structure [8, 9, 10] for which changes in entropy and Ricci curvature are positively correlated [11, 12]. In conjunction with the Fluctuation Theorem [13], we can conclude that an increase in Ricci curvature is positively correlated with an increase in network robustness, herein expressed as $\Delta \mathrm{Ric} \times \Delta R \geq 0$. This is the key observation that we will exploit below. This observation leads to a notion of curvature on the space of probability distributions on rather general metric measure spaces.

There have been several notions of graph curvature proposed, including those based on the Bochner-Weitzenböck decomposition of the Laplace operator [14] (Forman-Ricci curvature), and those based on Bakry-Émery theory [5, 15]. In this work, we use the formulation of Ollivier [16] because of its direct link

to the geometry of optimal mass transport, and therefore its link to entropy and network robustness. There are several other properties of the Ollivier-Ricci curvature that make it attractive for studying the robustness of networks. There is a direct correlation of changes in the rate function from large deviations theory and Ollivier-Ricci curvature [1, 16]. Second, the Ollivier-Ricci curvature is a natural measure of feedback connectivity. This is because the number of triangles in a network (redundant pathways) can be characterized by an explicit lower bound based on Ollivier-Ricci curvature [17]. Similar to control theory, redundancy in feedback is the main characteristic of a robust system architecture. Finally, positive Olliver-Ricci curvature governs the rate of convergence to an invariant distribution on a weighted graph which is modeled as a Markov chain. For these reasons, we chose to use the Ollivier-Ricci curvature for our analysis.

Ricci curvature and entropy

We briefly sketch the connection of curvature and entropy and curvature on a Riemannian manifold. Accordingly, let X denote a complete connected Riemannian manifold equipped with metric d. Ricci curvature provides a way of measuring the degree to which the geometry determined by a given Riemannian metric differs from that of ordinary Euclidean space [18]. The Ricci curvature tensor is obtained as the trace of the sectional curvature [18], and a lower bound on Ricci curvature provides an estimate of the tendency of geodesics to converge or diverge. Through the work of Lott-Sturm-Villani [11, 12], optimal transport connects the bounds on Ricci curvature with entropy, as sketched out below.

First, let $\mathscr{P}(X)$ denote the space of probability densities with finite second moments on X. One can show that $\mathscr{P}(X)$ also has a Riemannian structure [8, 10] via the Wasserstein metric W_2 from optimal mass transport theory (OMT). Next, for a probability measure $\mu \in \mathscr{P}(X)$, the *Boltzmann entropy* is defined as

$$H(\mu) = -\int_X \mu \log \mu \; \mathrm{dvol}.$$

Lott, Sturm, and Villani [11, 12] found a very interesting connection between Ricci curvature and the Boltzmann entropy via OMT. Namely, the Ricci curvature $\mathrm{Ric} \geq k$ if and only if the entropy functional is displacement k-concave along the 2-Wasserstein geodesics, that is, for $\mu_0, \mu_1 \in \mathscr{P}(X)$ we have

$$H(\mu_t) \geq (1-t)H(\mu_0) + tH(\mu_1) + k\frac{t(1-t)}{2} W_2(\mu_0,\mu_1)^2 \quad \forall t \in [0,1], \tag{1}$$

where $(\mu_t)_{0\leq t\leq 1}$ is a 2-Wasserstein geodesic between μ_0 and μ_1. This inequality indicates the positive correlation between changes in entropy and changes in curvature, which we denote as

$$\Delta H \times \Delta \mathrm{Ric} \geq 0.$$

Entropy and robustness

As previously mentioned, we are interested in the connection between robustness and the network entropy. Informally, functional robustness is defined as the ability of a system to adapt to random perturbations in its environment. This has been exploited in several works [1, 2, 3, 4] in order to employ changes in curvature as a proxy for robustness. This has been extensively explored in the aforementioned papers, and so we will only briefly describe the necessary results.

The starting point is the paper [13], in which the authors characterize robustness via the fluctuation decay rate after random perturbations. Denote by $p_\varepsilon(t)$, the probability that the deviation of the sample mean is more than ε at time t from the original value. Following large deviations theory [19], the *fluctuation decay rate* is defined as

$$R := \lim_{t\to\infty, \varepsilon\to 0} \left(-\frac{1}{t}\log p_\varepsilon(t)\right).$$

The larger the value of R, the faster the systems converges to a stationary state. The Fluctuation Theorem [13] states that the changes in entropy and robustness are positively correlated:

$$\Delta H \times \Delta R \geq 0. \tag{2}$$

Combining (1) and (2) give a relationship which indicates the correlation of changes in curvature and robustness:

$$\Delta R \times \Delta \text{Ric} \geq 0. \tag{3}$$

Thus changes in curvature serve as a measure of changes in system robustness. Positive changes indicate increase in robustness, while negative changes indicate the increase in system fragility.

Wasserstein distance on discrete spaces

We will not need a general definition of the Wasserstein distance in defining the curvature, and so we will suffice with a definition that applies to a weighted connected undirected graph $G = (V, E)$ with n nodes (V) and m edges (E). See [6, 7, 20] and the references therein for the details.

Given two probability densities $\rho^0, \rho^1 \in \mathbb{R}^n$ on the graph, the Kantorovich formulation of the optimal transport problem [6, 7] seeks a joint distribution $\rho \in \mathbb{R}^{n \times n}$ with marginals ρ^0 and ρ^1 minimizing the total cost $\sum c_{ij}\rho_{ij}$:

$$W_C(\rho^0, \rho^1) = \min_{\rho} \left\{ \sum_{i,j=1}^{n} c_{ij}\rho_{ij} \;\middle|\; \sum_k \rho_{ik} = \rho_i^0,\ \sum_k \rho_{kj} = \rho_j^1,\ \forall i, j \right\}. \tag{4}$$

Here, c_{ij} is the cost of moving unit mass from node i to node j. If c_{ij} is defined via some intrinsic distance on G (e.g., the hop metric), the minimum of (4) defines a metric W_1 (the Earth Mover's Distance) on the space probability densities on G.

An alternative formulation may be formulated via the fluxes $u \in \mathbb{R}^m$ on the edges. Letting $D \in \mathbb{R}^{n \times m}$ denote the oriented incidence matrix of $\mathcal{G}$, we have

$$W_1(\rho^0, \rho^1) = \min_u \left\{ \sum_{i=1}^{m} |u_i| \;\middle|\; \rho^0 - \rho^1 - Du = 0 \right\}.$$

The incidence matrix $D = [d_{ik}] \in \mathbb{R}^{n \times m}$ is defined by associating an orientation to each edge $e_k = (i, j) = (j, i)$ of the graph: one of the nodes i, j is defined to be the head and the other the tail, and then we set $d_{ik} = +1(-1)$ if i is the head (tail) of e_k and 0 otherwise. Compared to the Kantorovich formulation which has n^2 variables, the above formulation has only m variables. It may greatly reduce the computational load when the graph G is sparse, i.e., $m << n^2$ and is often the case in real data.

Olliver-Ricci curvature

In this section, we define the Ricci curvature on discrete networks. Specifically, we assume that our network is presented by an undirected and positively weighted graph, $G = (V, E)$, where V is the set of n vertices (nodes) in the network and E is the set of all edges connecting them, with $w_{xy} > 0$ denoting the weight of the edge between node x and y. (If there is no edge connecting the two nodes, then $w_{xy} = 0$.) We should note that in our treatment above we connected curvature and entropy via W_2, and now we will be using W_1. This technical point is discussed and justified in detail in [3].

Let $d_x := \sum_z w_{xz}$, where the sum is taken over all nodes z in a neighborhood of x. At each node, we define the probability measure

$$\mu_x(y) := \frac{w_{xy}}{d_x}.$$

Let $d : V \times V \to \mathbb{R}^+$ denote an intrinsic metric on G, e.g., the hop distance. Then for any two distinct points $x, y \in V$, the *Ollivier-Ricci (OR) curvature* [16] is defined as

$$k(x,y) := 1 - \frac{W_1(\mu_x, \mu_y)}{d(x,y)}.$$

Using this edge based notion of curvature, we can define the *scalar curvature* at a given node by

$$\hat{S}_{OR}(x) := \sum_y \mu_x(y) k(x,y), \tag{5}$$

where the sum is taken over all neighbors of x.

Given our above arguments, the positive correlation of robustness and curvature, we use curvature as a proxy for robustness in our study of autism. Curvature can be defined either *nodally* (scalar) or relative to *edges* (Ricci). Various advantages of using Ricci curvature in this framework are described in more detail in [1].

Supplemental Figures

For the following figures, VABS: Vineland Adaptive Behavior Scales-II, EOW: Expressive One-Word Picture Vocabulary Test, CGI: Clinical Global Impression Scales, TP1: time point 1 (initial clinical visit), TP2: time point 2 (six months after the infusion).

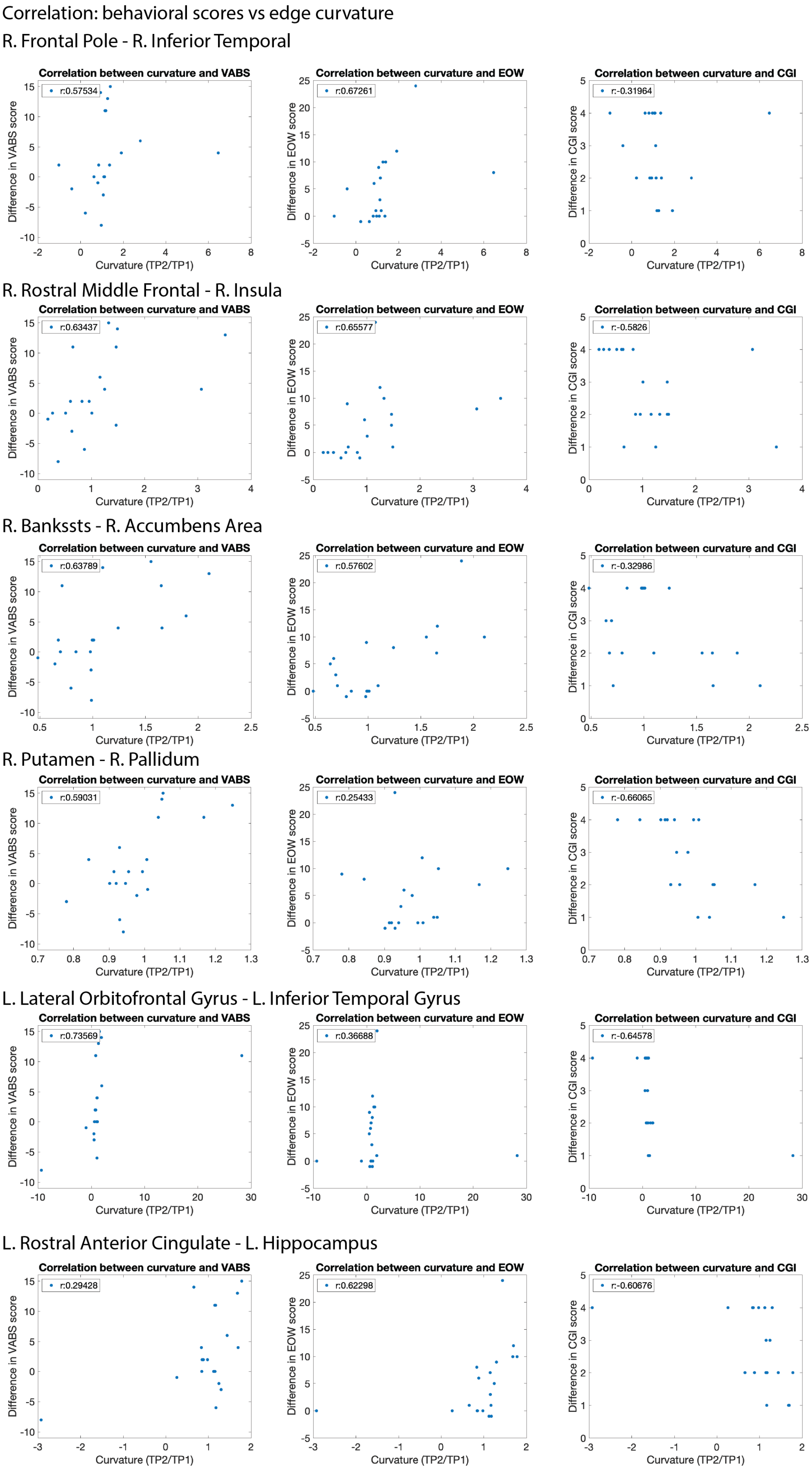

Figure 1. **Correlation between the ratio of edge curvature and difference in clinical scores**

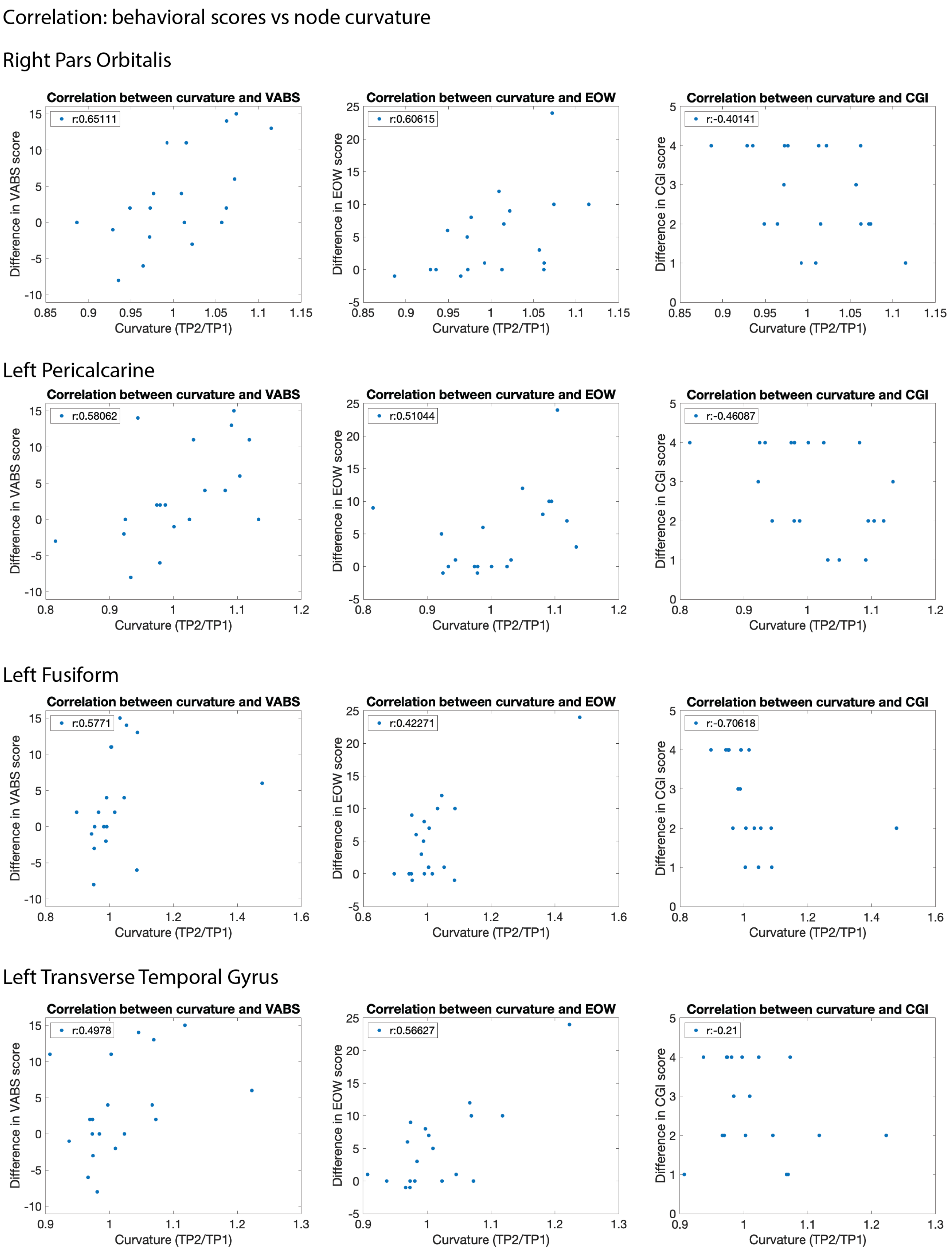

Figure 2. **Correlation between the ratio of node curvature and difference in clinical scores**

References

1. Sandhu, R. S. *et al.* Graph curvature for differentiating cancer networks. *Sci. Reports* **5**, 1–13, DOI: 10.1038/srep12323 (2015).
2. Sandhu, R. S., Georgiou, T. T. & Tannenbaum, A. R. Ricci curvature: An economic indicator for market fragility and systemic risk. *Sci. advances* **2**, e1501495 (2016).
3. Farooq, H., Chen, Y., Georgiou, T. T., Tannenbaum, A. & Lenglet, C. Network curvature as a hallmark of brain structural connectivity. *Nat. Commun.* **10**, 1–11, DOI: 10.1038/s41467-019-12915-x (2019).
4. Pouryahya, M. *et al.* Bakry-Émery Ricci curvature on weighted graphs with applications to biological networks. In *Int. Symp. on Math. Theory of Net. and Sys*, vol. 22 (2016).
5. Bakry, D. & Emery, M. Diffusions hypercontractives. *Lect. Notes Math. Seminaire de Probab. XIX 1983/84* 177–206, DOI: 10.1007/bfb0075847 (1985).
6. Rachev, S. T. & Rüschendorf, L. *Mass Transportation Problems: Volume I: Theory*, vol. 1 (Springer Science & Business Media, 1998).
7. Villani, C. *Topics in Optimal Transportation*. 58 (American Mathematical Soc., 2003).
8. Benamou, J.-D. & Brenier, Y. A computational fluid mechanics solution to the Monge-Kantorovich mass transfer problem. *Numer. Math.* **84**, 375–393 (2000).
9. Jordan, R., Kinderlehrer, D. & Otto, F. The variational formulation of the Fokker–Planck equation. *SIAM journal on mathematical analysis* **29**, 1–17 (1998).
10. Otto, F. The geometry of dissipative evolution equations: the porous medium equation. *Commun. Partial. Differ. Equations* (2001).
11. Lott, J. & Villani, C. Ricci curvature for metric-measure spaces via optimal transport. *Annals Math.* 903–991 (2009).
12. Sturm, K.-T. & Others. On the geometry of metric measure spaces. *Acta mathematica* **196**, 65–131 (2006).
13. Demetrius, L. & Manke, T. Robustness and network evolution—an entropic principle. *Phys. A: Stat. Mech. its Appl.* **346**, 682–696, DOI: 10.1016/j.physa.2004.07.011 (2005).
14. Forman, R. Bochners method for cell complexes and combinatorial ricci curvature. *Discret. Comput. Geom.* **29**, 323–374, DOI: 10.1007/s00454-002-0743-x (2003).
15. Klartag, B., Kozma, G., Ralli, P. & Tetali, P. Discrete curvature and abelian groups. *Can. J. Math.* **68**, 655–674, DOI: 10.4153/cjm-2015-046-8 (2016).
16. Ollivier, Y. Ricci curvature of metric spaces. *Comptes Rendus Math.* **345**, 643–646, DOI: 10.1016/j.crma.2007.10.041 (2007).
17. Bauer, F., Jost, J. & Liu, S. Ollivier-Ricci curvature and the spectrum of the normalized graph Laplace operator. *Math. Res. Lett.* **19**, 1185–1205 (2012).

18. Do Carmo, M. P. *Riemannian Geometry* (Birkhauser, 2015).

19. Varadhan, S. R. S. *Large Deviations and Applications* (Society for Industriel and Applied Mathematics, 1984).

20. Cedric, V. *Optimal Transport: Old and New* (Springer, 2009).
